# Vascular regeneration and skeletal muscle repair induced by long-term exposure to SDF-1α derived from engineered mesenchymal stem cells after hindlimb ischemia

**DOI:** 10.1038/s12276-023-01096-9

**Published:** 2023-10-02

**Authors:** Jin-Ju Kim, Jae-Hyun Park, Hyeok Kim, Woo-Sup Sim, Seokbeom Hong, Yeon-Jik Choi, Hyo-Jin Kim, Soon Min Lee, Dongha Kim, Sun-woong Kang, Kiwon Ban, Hun-Jun Park

**Affiliations:** 1 Department of Biomedicine & Health Sciences, The Catholic University of Korea, Seoul, South Korea; 2 Division of Cardiology, Department of Internal Medicine, Seoul St. Mary’s Hospital, College of Medicine, The Catholic University of Korea, Seoul, South Korea; 3 Department of Thoracic and Cardiovascular Surgery, Seoul St. Mary’s Hospital, College of Medicine, The Catholic University of Korea, Seoul, South Korea; 4 Division of Cardiology, Department of Internal Medicine, Eunpyeong St. Mary’s Hospital, College of Medicine, The Catholic University of Korea, Seoul, South Korea; 5 SL BIGEN, Inc., Kyonggi-do, South Korea; 6 Department of Anatomy, College of Medicine, The Catholic University of Korea, Seoul, South Korea; 7 Research Group for Biomimetic Advanced Technology, Korea Institute of Toxicology 7 (KIT), Daejeon, South Korea; 8 Department of Biomedical Sciences, City University of Hong Kong, Kowloon Tong, Hong Kong

**Keywords:** Regeneration, Peripheral vascular disease

## Abstract

Despite recent progress in medical and endovascular therapy, the prognosis for patients with critical limb ischemia (CLI) remains poor. In response, various stem cells and growth factors have been assessed for use in therapeutic neovascularization and limb salvage in CLI patients. However, the clinical outcomes of cell-based therapeutic angiogenesis have not provided the promised benefits, reinforcing the need for novel cell-based therapeutic angiogenic strategies to cure untreatable CLI. In the present study, we investigated genetically engineered mesenchymal stem cells (MSCs) derived from human bone marrow that continuously secrete stromal-derived factor-1α (SDF1α-eMSCs) and demonstrated that intramuscular injection of SDF1α-eMSCs can provide long-term paracrine effects in limb ischemia and effectively contribute to vascular regeneration as well as skeletal muscle repair through increased phosphorylation of ERK and Akt within the SDF1α/CXCR4 axis. These results provide compelling evidence that genetically engineered MSCs with SDF-1α can be an effective strategy for successful limb salvage in limb ischemia.

## Breakthrough in limb ischemia: the power of genetically modified MSCs

Researchers have developed a strategy using SDF1α-engineered mesenchymal stem cells (SDF1α-eMSCs) to simultaneously regenerate blood vessels and skeletal muscles in limbs experiencing ischemia. In a mouse model of hindlimb ischemia, intramuscular injection of SDF1α-eMSCs improved blood perfusion, preserved limb function, and prevented amputation. In vitro result showed that SDF1α-eMSCs not only amplified the angiogenic potential and survival of endothelial cells, but also demonstrated enhanced survival and migration abilities in myoblasts. The study suggests that SDF1α-eMSCs can be considered an effective therapeutic strategy for limb salvage in patients with critical limb ischemia.

## Introduction

Peripheral artery disease (PAD) is one of the leading causes of atherosclerotic cardiovascular mortality, with a prevalence of more than 20% in individuals aged 50 years or older^1^. PAD has alarmingly high rates of morbidity and mortality, with approximately 40% of patients at risk of amputation or death within a short time^2,3^. Critical limb ischemia (CLI), the end stage of lower extremity PAD, severely obstructs blood flow to the lower limb, resulting in ischemic rest pain, ulcers, gangrene, and a substantial risk of limb loss^4^. Revascularization is the preferred therapeutic strategy in patients with PAD; however, despite recent progress in medical and endovascular therapy of patients with CLI, approximately 20–30% of patients with CLI are not considered candidates for revascularization therapy, and the prognosis of patients with advanced PAD remains poor^5^.

Indeed, CLI severely damages not only blood vessels but also skeletal muscles, which is another critical component of the limb due to poor nutrient supply, and thus, it negatively contributes to amputation and eventual limb loss^6,7^. Nevertheless, current therapeutic regimens for treating PAD are focused only on therapeutic angiogenesis by the induction of blood reflow in ischemic limbs through the delivery of growth factors/proteins, genes, or various types of stem cells to ischemic tissues^8,9^. Despite extensive efforts to induce therapeutic angiogenesis, to date, satisfactory rescue of CLI patients from major limb amputation has not been achieved, suggesting that vascular regeneration alone is insufficient to achieve complete limb salvage^10,11^.

Stromal cell-derived factor-1 (SDF-1α) is a chemokine that plays a major role in the trafficking and homing of CXCR4+ cells^12^. During injury, cells from the injured organ highly express SDF-1α, which leads to the recruitment and retention of CXCR4+ cells at the injury site in response to chemotactic attraction toward the SDF-1α gradient^13^. Several previous studies have reported that SDF-1α expression was also elevated in ischemic skeletal muscle fibers from patients with CLI^14^. As such, SDF-1α may act as a proangiogenic factor, recruiting CD34+ endothelial progenitor cells and inducing vascular regeneration via induction of vasculogenesis^15–17^. Furthermore, it has been reported that both muscle progenitor cells (satellite cells) and myoblasts, which are required for muscle generation, are CXCR4+ cells, and these cells are able to migrate to damaged areas in response to an SDF-‍1α gradient and are therefore expected to have therapeutic potential in muscle regeneration^14,18^. Therefore, SDF-1a is presumed to play a vital role in the regeneration of both blood vessels and skeletal muscle in damaged ischemic tissues. Despite the many beneficial effects of SDF-1α, its widespread use as a therapeutic agent has critical limitations. SDF-1α has a short half-life since it undergoes rapid proteolysis in a protease-rich tissue environment^19^. Therefore, therapeutic SDF-1α should be injected repeatedly for effective limb salvage, which raises safety concerns about side effects such as toxicity and inflammation^20^.

Therefore, in the present study, we developed a strategy to concurrently rejuvenate both blood vessels and skeletal muscles in limbs undergoing ischemia. We reasoned that since the limb is composed of skeletal muscle and blood vessels, therapeutic strategies for treating ischemic limbs should be focused on comprehensively repairing both tissues together to accomplish effective limb repair. To achieve this aim, we selected SDF-1α as a target therapeutic molecule and tested it in a mouse model of hindlimb ischemia (HLI). To overcome the short half-life of SDF-1α, we engineered mesenchymal stem cells capable of continuously secreting SDF-1α (SDF1α-eMSCs). We hypothesized that intramuscular injection of SDF1α-eMSCs could promote the regeneration of both blood vessels and skeletal muscle simultaneously as a result of steady secretion of SDF-1α and other beneficial paracrine factors within the hindlimb. The results demonstrated that implantation of SDF1α-eMSCs not only improved the number of blood vessels through the recruitment of CXCR4+ vasculogenic cells but also competently promoted skeletal muscle repair with the enhancement of myogenesis, as evidenced by increased numbers of newly regenerated myofibers and cross-sectional area of myofibers. These results therefore provide compelling evidence that SDF-1α-eMSCs can be considered an effective therapeutic strategy for limb salvage in CLI patients.

## Materials and methods

### Cell culture

BM-MSCs were obtained from the Catholic Institute of Cell Therapy (CIC; Catholic MASTER Cells, CIC, Korea). Human bone marrow aspirates were obtained from the iliac crest of healthy donors aged 20 to 55 years with approval from the Institutional Review Board of Seoul St. Mary’s Hospital (approval numbers KIRB-00344-009 and KIRB-00362-006). Bone marrow aspirates from each donor who consented were collected and sent to the good manufacturing practice (GMP)-compliant facility of the CIC (Seoul, Korea; www.cic.re.kr) for isolation, expansion, and quality control of human BM-MSCs. The MSC growth medium was low-glucose Dulbecco’s modified Eagle’s medium (DMEM, Gibco) with 7% fetal bovine serum (FBS; Gibco), 15 ng/μL IGF-1 and 125 pg/μL bFGF. BM-MSCs were added to 100-mm tissue culture dishes (TPP) and incubated in growth medium at 37 °C with 5% CO_2_. The medium was replaced every other day. The cells were detached when they reached 70 to 90% confluence and were replated at a density of 5 × 10^3^ cells/cm^2^. The cells were expanded through two to four passages at the GMP-compliant facility. Human umbilical vein endothelial cells (HUVECs) were obtained from Promocell and cultured in EGM-2 complete medium (Lonza). The HUVECs were plated into 100-mm tissue culture dishes (TPP) at a density of 2.5 × 10^3^ cells/cm^2^ and incubated at 37 °C with 5% CO_2_. The media were replaced every other day. C2C12 cells were obtained from ATCC and cultured in 100-mm tissue culture dishes (TPP) at a density of 5 × 10^3^ cells/cm^2^. The C2C12 growth medium consisted of high glucose Dulbecco’s modified Eagle’s medium (DMEM, Gibco) with 10% fetal bovine serum (FBS; Gibco), and the cells were incubated at 37 °C with 5% CO_2_.

### Generation of engineered SDF1α-eMSCs

BM-MSCs (Catholic MASTER Cells) were obtained from the Catholic Institute of Cell Therapy (CIC, Seoul, Korea). For immortalized MSCs, replication-incompetent lentiviral vectors containing hTERT and c-Myc were prepared and transfected into the cells. For immortalized MSCs expressing SDF-1α, replication-incompetent lentiviral vectors expressing human SDF-1α were prepared and then transfected into immortalized MSCs. Then, SDF1α-eMSCs were isolated as a monoclonal cell population using the limiting dilution method. The final monoclonal cells were selected based on SDF-1α protein secretion, proliferation rate, and other MSC phenotypes. The SDF1α-eMSCs were cultured in low-glucose Dulbecco’s modified Eagle’s medium (DMEM, Gibco) with 10% fetal bovine serum (FBS, Gibco), 10 ng/mL basic fibroblast growth factor (bFGF, Peprotech) and 2 μg/mL doxycycline (Clontech) at 37 °C and 5% CO_2_.

### Production of conditioned medium (CM)

BM-MSCs and SDF1α-eMSCs were cultured at 37 °C with 5% CO_2_, and when the cultures became confluent, the culture media was removed, rinsed with DPBS, and then replaced with serum-free media supplemented with 1% Gibco™ Antibiotic-Antimycotic. The culture was maintained for 72 h. After 72 h, the medium was collected and centrifuged at 1500 rpm for 10 min to remove cell debris. The supernatant was harvested and stored at −70 °C.

### Protection assay (FACS)

When HUVECs and C2C12s reached 100% confluence, the medium was replaced with EBM or serum-free DMEM to mimic starvation conditions. Cells cultured in EGM (HUVECs) or 10% FBS + high glucose DMEM (C2C12) were the positive control (normal group), and those cultured in EBM (HUVECs) or serum-free DMEM (C2C12) were the negative control (control group). Fifty percent of each BM CM and SDF1α CM sample was added to 50% EBM or serum-free media (BM CM group, SDF1α CM group). After 48 h in starvation culture, flow cytometric analyses were performed using a Membrane Permeability/Dead Cell Apoptosis Kit with YO-‍PRO™-1 and propidium iodide (PI) (Invitrogen, V13243). The cells (1 ×10^6^ cells/mL) were stained with 0.1 µM YO-PRO™-1 and 1.5 µM PI. After incubation on ice for 30 min, the cell fluorescence was analyzed using flow cytometry (BD FACS).

### Viability/cytotoxicity assay

Viability and cytotoxicity were assessed using a LIVE/DEAD™ Viability/Cytotoxicity Kit (Invitrogen, L3224) according to the manufacturer’s instructions. The medium was exchanged with phenol red-free DMEM with a calcein and ethidium homodimer-1 dye mixture and incubated for 30 min; then, the labeled cells were analyzed under a fluorescence microscope (Nikon).

### Migration assay

HUVECs (5 × 10^4^) were seeded onto the upper layer of a Transwell™ insert (8-μm pore) with EBM. The inserts were placed in a 24-well plate containing the test media (EGM2, EBM, BM CM, or SDF1α CM). The C2C12 cells (5 × 10^4^) were seeded in the upper layer of a Transwell™ insert (8-μm pore) with serum-free DMEM-high glucose and placed in a 24-well plate containing the test media; the normal group had EGM (HUVECs) and 10% FBS+high glucose DMEM (C2C12), the control group had EBM (HUVEC) or serum free media (C2C12), the BM group had 50% serum free media + 50% BM CM, and the SDF1α group had 50% serum free media + 50% SDF1α CM. After 16 h of culture, the cells were fixed with 4% PFA for 10 min, and staining was performed using 0.1% crystal violet (Sigma-Aldrich) for 10 min. After the Transwell™ membrane was rinsed with distilled water, the upper side of the membrane was gently wiped with a cotton swab to remove nonmigrated cells. The migrated cells were counted using a light microscope, and the stained area of the membrane was calculated using ImageJ software.

### Tube formation assay

Matrigel growth factor reduced base membrane matrix (Corning, 354230) was added to each well of µ-slide (ibidi). HUVECs were stained with 1 µM cell tracker (Invitrogen, C7000), and then, cells (3 × 10^5^/ml) were added onto Matrigel-coated wells containing media (normal group: EGM, control group: EBM, BM CM group: 50% EBM + 50% BM CM, SDF1α CM group: 50% EBM + 50% SDF1α CM) and incubated at 37 °C with 5% CO_2_ for 8 h. After 8 h, the cells were visualized using a light microscope, and the formation of tube structures was calculated using ImageJ software.

### AMD3100 treatment

Pretreatment with AMD3100 (Selleckchem, S3013) was performed before the assay. The cells were treated at a concentration of 10 μM in serum-free media (EBM or high glucose DMEM) overnight prior to conducting each assay.

### Western blotting

Cell lysates were prepared in RIPA buffer (Thermo Fisher Scientific, 89901) with 0.5 M EDTA (Thermo Fisher Scientific, 78440) and phosphatase inhibitor cocktail (Thermo Fisher Scientific, 78440). Total cell protein was quantified using the Pierce™ BCA Protein Assay Kit (Thermo Fisher Scientific, 23225). Equal amounts of total protein sample were taken, and Fluorescent-Compatible Sample Buffer (Invitrogen, LC2570) with 10X Bolt™ Sample Reducing Agent (Invitrogen, B0009) was added. Next, each sample was heated to 80 °C for 15 min and then centrifuged at 16,000 × g for 3 min at 4 °C. The supernatant was collected for western blotting. Equal amounts of loaded protein were resolved by NuPage™ 4 to 12% Bis-Tris Protein Gel (Invitrogen, NP0321BOX) in each experiment. Next, proteins were transferred to iBlot™ NC Transfer Stacks (Invitrogen, IB301002), and membranes were incubated with primary antibody overnight at 4 °C and the secondary antibody for 1 h at room temperature. For quantification of target signal expression, all samples to be compared were run on the same gel and imaged. Total protein normalization was carried out using No-Stain™ Protein Labeling Reagent (Invitrogen, A44717) according to the manufacturer’s instructions. Bands were quantified using an iBright™ FL1500 Imaging System (Invitrogen, A44115). The antibodies used in western blotting were anti-Erk1/2 (Cell Signaling, #4696), anti-phospho Erk1/2 (Cell Signaling, #4376), anti-Akt (Cell Signaling, #4693), anti-phospho Akt (Cell Signaling, #4051), anti-HSP 90α/β (Santa Cruz, sc-13119), donkey anti-mouse IgG Alexa Fluor Plus 680 (Abcam, ab175774) and donkey anti-rabbit IgG Alexa Fluor Plus 800 (Abcam, ab216773).

### Hindlimb ischemia model

All animal studies were approved by the Institutional Animal Care and Use Committee (IACUC) of The Catholic University of Korea (Approval number: CUMC-2021-0056-02). IACUC and the Department of Laboratory Animals (DOLA) at The Catholic University of Korea, Songeui Campus accredited the Korea Excellence Animal Laboratory Facility at the Korea Food and Drug Administration in 2017, and the facility acquired full AAALAC International accreditation in 2018. All animal procedures conformed to the guidelines from Directive 2010/63/EU of the European Parliament addressing the protection of animals used for scientific purposes or the NIH guidelines. Balb/c nude mice (8 weeks old, male, 20–25 g) were procured from Orientbio, Korea, and were anesthetized with 2% isoflurane. Body temperature was maintained on a 37 °C heating pad to prevent cooling during the procedure. Ischemia was induced by ligating the proximal superficial epigastric artery and its bifurcation into the common femoral artery, occluding the distal and proximal ends of the femoral artery with double-knotted sutures (7-0 silk), and the intervening 2–3 mm of the artery was excised. Subsequently, cells (1 × 10^6^) were injected into two intramuscular sites of the medial hindlimb at a volume of 20 µL per shot. The experimental groups were (1) control (PBS), (2) BM-MSCs, and (3) SDF1α-eMSCs. Additionally, mice received ketoprofen (2 mg/kg) intraperitoneally for three days following the surgery.

### Blood flow and limb salvage measurements

For evaluation of regenerative efficacy, animals were tracked by serial monitoring of hind limb blood perfusion using a laser Doppler perfusion imaging system (Omegawave, Japan) at Days 0, 7, 14, 21 and 28 post-surgery. The blood flow from the knee joint to the toe region was quantified by analyzing color-coded digital images, and the perfusion rate was calculated. In addition, at Day 28 post-implantation, the percentages of five statuses (limb loss, foot necrosis, tip necrosis, toe necrosis or limb salvage) were quantified. The limb loss score was graded as whole limb loss (5), limb loss (4), foot necrosis (3), tip necrosis (2), toe necrosis (1) or limb salvage (0).

### Determination of fibrosis

Masson’s trichrome (MT) staining (Sigma, St. Louis, MO, USA) was performed to determine the area of fibrotic tissue in the ischemic hind limb. Briefly, three paraffin slides were preincubated in a 37 °C dry oven before deparaffinization and rehydration. The paraffin sections were then refixed for 1 h in 56 °C Bouin’s solution. These sections were stained using Weigert’s iron hematoxylin solution for 15 min at room temperature and further stained with Biebrich scarlet-acid fuchsin solution for 20 min at room temperature. Finally, the sections were counterstained with aniline blue for 15 min, followed by incubation in 1% acetic acid for 1 min at room temperature. Extensive washes were performed between each step. The collagen fibers appeared blue, and viable skeletal muscle appeared red. Imaging of the tissue sections was performed with a slide scanner (Pannoramic MIDI). All other items, including the fibrotic area, were quantified using ImageJ software.

### Immunohistochemistry analyses

At the time of sacrifice, the limb tissues were fixed in 4% paraformaldehyde overnight, and then, paraffin blocks were made. The tissue was sectioned into 5 μm cross sections starting at the top of the apex using a microtome (Leica, RM2255, Germany). The sections were stored at −20 °C before use. After deparaffinization and rehydration, antigen retrieval with target retrieval solution (DAKO) was performed in a humid chamber. The sections were blocked and incubated with diluted primary antibody (Dako) at 4 °C overnight. The primary antibodies used in this study were mouse anti-CD31 (Novus, AF3628; 1:200), rabbit anti-αSMA (Abcam, ab5617; 1:200), rabbit anti-laminin (Sigma, L9393; 1:200), mouse anti-CD68 (Abcam, ab31630; 1:200), rabbit anti-CD206 (Abcam, ab64693; 1:200) and rabbit anti-iNOS (Abcam, ab15323; 1:200). After three washes with 1% Tween^®^ 20 in PBS, the samples were incubated with secondary antibody for 90 min at room temperature in the dark. The secondary antibodies used in this study were anti-goat Alexa Fluor 488 (Invitrogen; 1:400) and anti-mouse Alexa Fluor 647 (Invitrogen; 1:400). After three washes with PBS, the sections were stained with DAPI solution (VectaShield) for nuclear staining and then mounted on slides. Quantification was performed for five random microscopic fields using a fluorescence microscope (Nikon) and was calculated using ImageJ. Each image was used for statistical analysis.

### Statistical analyses

All data are presented as the mean ± standard error of the mean (SEM). The statistical significance (*P* < 0.05) was determined by a two-tailed t test using GraphPad Prism Software.

## Results

### SDF1α-eMSCs enhanced the angiogenic potential of endothelial cells and their survival in vitro

**Fig. 1:**
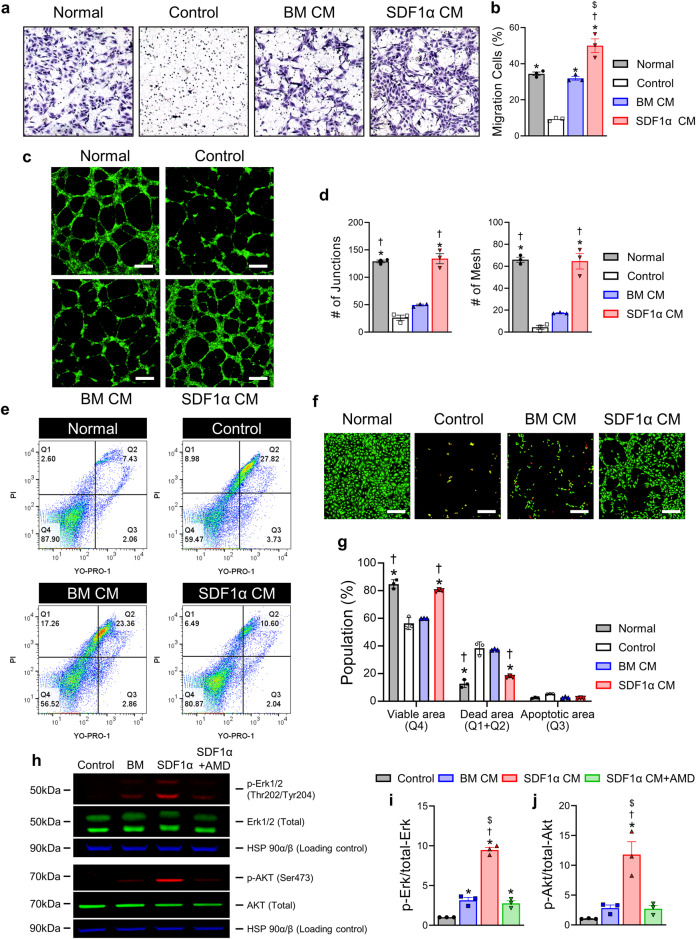
The paracrine effects of SDF1α-eMSCs on endothelial cells. HUVECs were incubated in media appropriate for each group to evaluate their capability (detailed information in the Methods). **a**, **b** Endothelial cell (EC) migration assay. **a** Representative images obtained under an inverted microscope and a quantification summary are shown. *p < 0.05 versus the control; †*p* < 0.05 versus BM CM; $*p* < 0.05 versus normal; *n* = 3 per group. **c**, **d** Tube formation assay. HUVECs cultured on Matrigel™ were incubated with 50% SDF1α CM to examine the vasculogenic potential. Representative images of tubes formed on Matrigel™ and quantification summary. Scale bar = 200 μm. *p < 0.05 versus the control; †*p* < 0.05 versus BM CM; *n* = 3 per group. **e**–**g** Protection assay under starvation conditions. Incubation with the appropriate culture media for each group for two days as determined by the YO-PRO™/PI and LIVE/DEAD™ assays. Scale bar = 200 μm. *p < 0.05 versus the control; †*p* < 0.05 versus BM CM; *n* = 3 per group. **h**–**j** Representative images of western blot analyses (anti-Erk1/2, anti-pErk1/2 and anti-Akt, anti-pAkt). HUVECs were incubated with CM for 30 min after starvation overnight. *p < 0.05 versus the control; †*p* < 0.05 versus BM CM; $*p* < 0.05 versus SDF1α CM + AMD; *n* = 3 per group. Data are presented as the mean ± SEM.

In our previous study, we verified that SDF1α-eMSCs were indistinguishable from normal BM-MSCs with respect to their cell morphology and MSC markers (Supplementary Fig. 1a)^21^. However, we confirmed that SDF1α-eMSCs stably secreted human SDF-1α protein, as determined by a human SDF-1α enzyme-linked immunosorbent assay (ELISA)^21^. Therefore, we expected that the paracrine effects of SDF1α-eMSCs would be stronger than those of BM-MSCs in vitro. Initially, we evaluated the angiogenic potential of HUVECs pretreated with 50% conditioned media (CM) harvested from cultured SDF1α-eMSCs or BM-MSCs for 3 days. In EC migration assays, as shown in Fig. 1a, b, the addition of CM from SDF1α-eMSCs (SDF1α CM) significantly enhanced the migration of HUVECs compared with the migration of ECs treated with CM from BM-MSCs (BM CM), suggesting that cytokines released from SDF1α-eMSCs bolster the mobility of ECs. In addition, we performed Matrigel™ tube-formation assays, an experiment that mimics several critical steps in capillary tube formation and angiogenesis, such as proliferation, migration, and differentiation. The results from Matrigel™ tube formation assays demonstrated that the number of branches formed in the HUVECs treated with SDF1α CM was significantly greater than that in the HUVECs treated with BM CM (Fig. 1c, d). Next, we examined whether SDF1α CM could protect ECs from ischemic insult. To achieve this, we assessed the survival of ECs after exposing them to starvation culture conditions simulating ischemic injury in vitro using a Membrane Permeability/Dead Cell Apoptosis Kit with YO-‍PRO™-1 and propidium iodide. YO-PRO is a nuclear marker useful for detecting apoptotic cells. YO-PRO enters the intracellular space and can only bind to the DNA of apoptotic cells. However, since PI cannot pass through the cell membrane in live cells, PI can be used to detect dead cells. Dead cells with damaged cell membranes take up PI, leading to binding with intracellular DNA and emitting fluorescence. By flow cytometry analysis, we observed that the cell populations in Q1 + Q2 (positive for PI and YO-PRO: dead cells) and Q3 (positive for YO-PRO: apoptotic cells) in the SDF1α CM-treated ECs were significantly lower than those in the other experimental groups. In addition, cell populations in Q4 (negative for PI and YO-PRO: viable cells) in the SDF1α CM-treated ECs were substantially greater than those of the other experimental groups, confirming the protective effects of SDF1α CM (Fig. 1e–g, Supplementary Fig. 2). Activation of the ERK pathway promotes cell survival and migration^22^. We also confirmed that SDF1α CM treatment significantly increased the phosphorylation of Akt and extracellular signal-related kinase (ERK) in HUVECs, as determined by western blotting. However, increased phosphorylation of ERK and Akt by SDF1α CM was substantially abrogated by cotreatment with AMD3100 (CXCR4 inhibitor), suggesting that it was dependent on the SDF1α/CXCR4 axis (Fig. 1h–j). Altogether, these findings suggest that SDF1α-eMSCs can enhance the angiogenic potential of ECs as well as their survival in vitro under conditions that mimic ischemia.

### SDF1α-eMSCs facilitated the migration of myoblasts and their survival in vitro

**Fig. 2:**
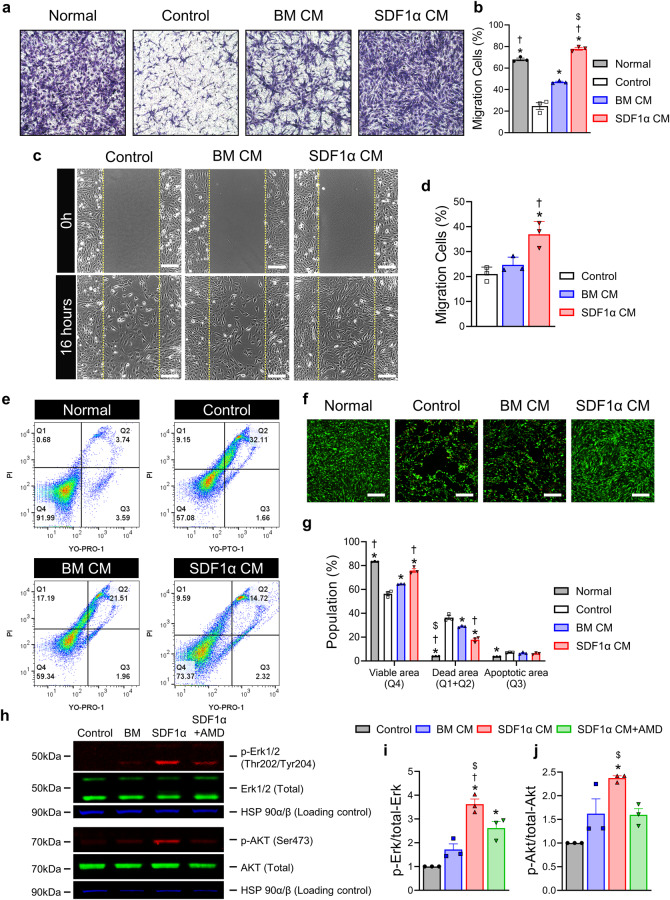
Paracrine effects of SDF1α-eMSCs on myoblasts. C2C12 cells were incubated in media appropriate for each group to evaluate their capability (detailed information in the Methods). **a**, **b** C2C12 migration assay. **a** Representative images obtained under an inverted microscope and (**b**) quantification summary are shown. *p < 0.05 versus the control; †*p* < 0.05 versus BM CM; $*p* < 0.05 versus normal; *n* = 3 per group. **c**, **d** Scratch assay (wound-healing assay). **c** Images were obtained immediately after the scratches had been made and then after 16 h of additional incubation in the presence of BM CM and SDF1α CM compared with serum-free media (control group). The yellow line indicates the border line. **d** Quantification summary is shown. Scale bar = 200 μm. *p < 0.05 versus the control; †*p* < 0.05 versus BM CM; *n* = 3 per group. **e**–**g** Protection assay under starvation conditions. Incubation with the appropriate culture media for each group for two days, as determined by the YO-PRO™/PI and LIVE/DEAD™ assays. **e** Representative images obtained under an inverted microscope and (**f**) quantification summary are shown. Scale bar = 200 μm. *p < 0.05 versus the control; †*p* < 0.05 versus BM CM; $*p* < 0.05 versus SDF1α CM; *n* = 3 per group. **h–j** Representative images of western blot analyses (anti-Erk1/2, anti-pErk1/2 and anti-Akt, anti-pAkt) of HUVECs. The cells were incubated with CM for 30 min after starvation overnight. *p < 0.05 versus the control; †*p* < 0.05 versus BM CM; $*p* < 0.05 versus SDF1α CM + AMD; *n* = 3 per group. Data are presented as the mean ± SEM.

Despite damage to the muscle at the ischemic site after ischemic injury, skeletal muscle has a robust regenerative capacity in response to injury^23^. Because myofiber regeneration requires the fusion of hundreds or thousands of myoblasts, the myogenic capacity depends on the recruitment of myoblasts^24^. Therefore, we evaluated whether the paracrine effects of SDF1α-eMSCs could increase the migratory capacity of myoblasts in vitro using the C2C12 cell line. Transwell™ migration and scratch assay results indicated that the migration of C2C12 cells increased significantly in the SDF1α CM treatment group compared with the control and BM CM groups (Fig. 2a–d). Next, we confirmed that SDF1α CM could increase the survival of myoblasts under starvation culture conditions, as previously demonstrated in HUVECs. Incubation with SDF1α CM significantly improved C2C12 cell survival compared with that of the starvation and BM CM groups (Fig. 2e-g). The effects on migration and survival were dependent on the activation of ERK and Akt signaling (Fig. 2h–j). Furthermore, to determine if these pathways had an impact on cell function, we treated the SDF1a CM group with AMD3100 in our previous experiments. As a result, the effects of SDF1a CM on cell function were reduced by treatment with AMD3100 (Supplementary Figs. 3, 4). The findings suggest that SDF1α-eMSCs can facilitate the migration of myoblasts as well as their survival in vitro under conditions that mimic ischemia.

### Intramuscular injection of SDF1α-eMSCs improved tissue perfusion and preserved limb function in hindlimb ischemia

**Fig. 3:**
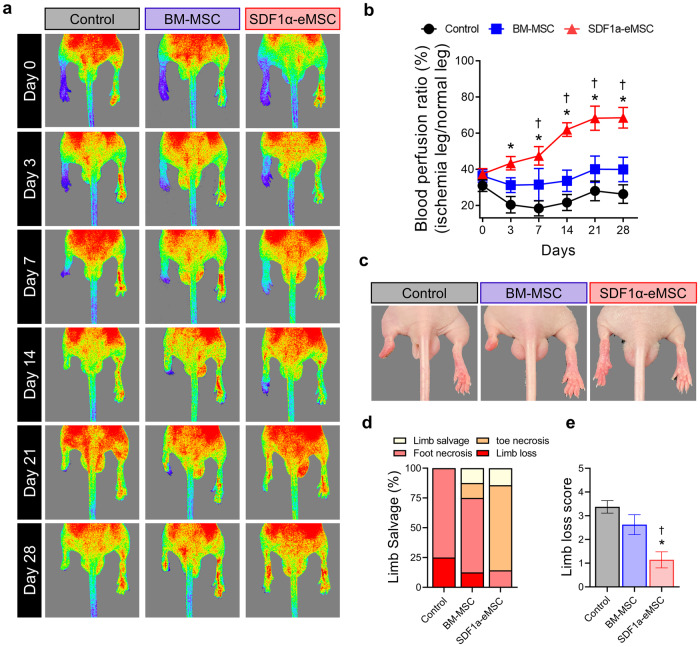
Therapeutic effects of SDF1α-eMSCs in a mouse model of hindlimb ischemia. **a** Laser Doppler perfusion imaging (LDPI) of the ischemic hindlimb at Days 0, 3, 7, 14, 21, and 28 after implantation. **b** Quantification of the blood perfusion ratio of the left ischemic limb compared with the nonischemic right limb in each group (*n* = 7–8). Statistical significance was determined using two-way ANOVA with Tukey post hoc pairwise comparisons; *p < 0.05 versus the control; †*p* < 0.05 versus BM-MSC group. **c** Representative optical image on Day 28. **d** Percentage of limb salvage in ischemic limb. **e** Scoring for the physiological status of ischemic limbs at Day 28 (0: limb salvage, 1: tip necrosis, 2: toe necrosis, 3: foot necrosis, 4: limb loss, 5: whole limb loss).

To investigate whether intramuscular injection of SDF1α-eMSCs could improve blood perfusion to ischemic limbs and preserve limb function without amputation, we induced limb ischemia by ligating the femoral artery, and SDF1α-eMSCs were transplanted intramuscularly in the center of the lower calf muscle. We first performed serial blood flow measurements using laser Doppler perfusion (LDPI) imaging over the course of 4 weeks postoperatively. Of interest, blood perfusion in the SDF1α-eMSC group increased gradually beginning on Day 3 and was significantly preserved for 4 weeks compared with that of the control and BM-MSC groups (Fig. 3a, b and Supplementary Fig. 5). Furthermore, the limb salvage rate and its quantification score were substantially higher in the SDF1α-eMSC group than in the control and BM-MSC groups, as estimated by the physiological status of the ischemic limbs 4 weeks after ischemic insult (Fig. 3c–e). These findings suggested that intramuscular injection of SDF1α-eMSCs improved blood perfusion and preserved limb function in the ischemic limbs of mice.

### SDF1α-eMSCs provide long-term paracrine effects on vascular regeneration by promoting angiogenesis

**Fig. 4:**
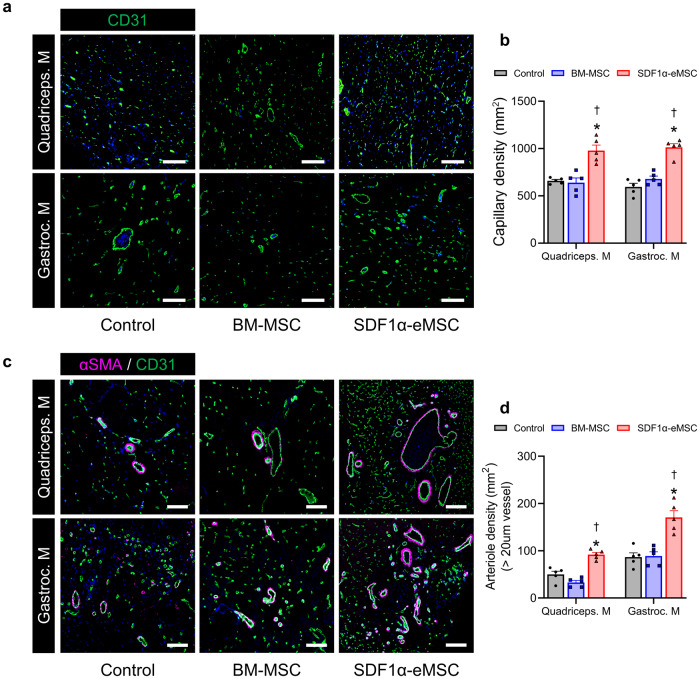
SDF1α-eMSCs significantly promote angiogenesis at Day 28 following hindlimb ischemia. **a** Representative image of quadriceps and gastrocnemius muscle capillaries stained for CD31 on Day 28 after HLI. **b** For quantification, the number of capillaries within five randomly selected fields in each ischemic muscle was counted. Scale bars, 100 μm. *p < 0.05 versus the control; †*p* < 0.05 versus BM-MSC group; *n* = 5 per group. **c** Representative immunostaining images with αSMA (violet), CD31 (green), and DAPI for nuclei (blue) at Day 28 after HLI and (**d**) the corresponding quantification summary. Scale bars, 100 μm. * *p* < 0.05 versus the control; † *p* < 0.05 versus BM-MSC group; *n* = 5 for each group. Data are presented as the mean ± SEM.

Next, we analyzed the paracrine effects of SDF1α-eMSCs on muscle regeneration through histological assessment. We performed CD31 staining to quantify the capillary density in cross-sectional tissue samples of quadriceps and gastrocnemius muscles according to the severity of ischemia^25^. The capillary density in both the quadriceps and gastrocnemius muscles in the SDF1α‍-eMSC group was significantly greater than that in the other experimental groups (Fig. 4a, b). Interestingly, the density of arterioles costained with αSMA and CD31 was significantly greater in the SDF1α-eMSC group than in the other groups, suggesting that SDF1α-eMSCs played an important role in arterial reassembly during the ingrowth of the alternative collateral artery network (Fig. 4c, d).

### SDF1α-eMSCs contributed to skeletal muscle repair by enhancing myogenesis and reducing fibrofatty infiltration

**Fig. 5:**
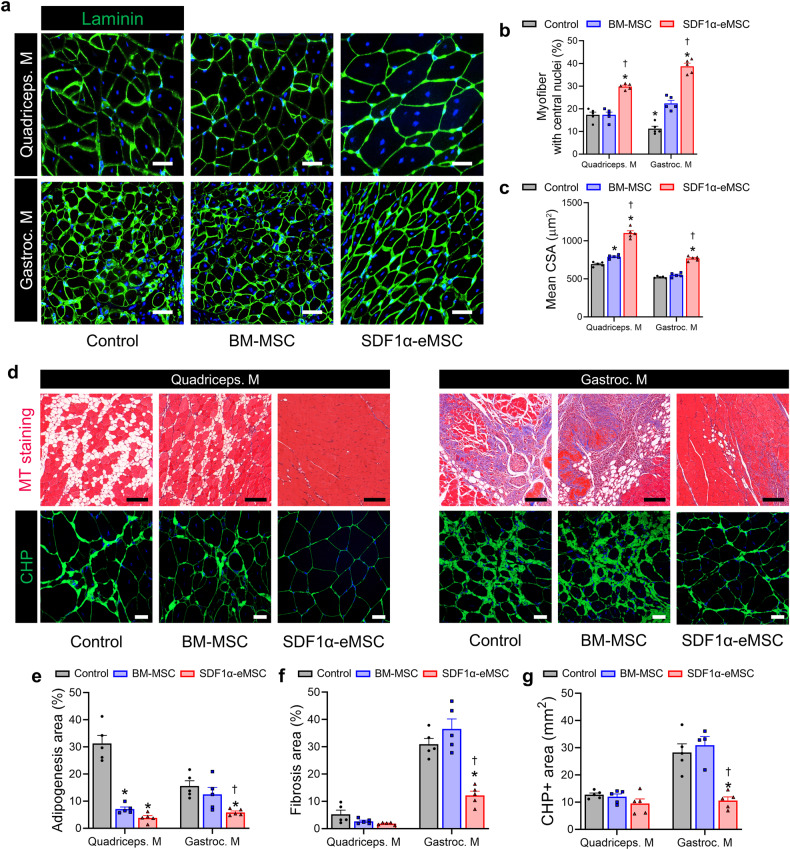
SDF1α-eMSCs increase the regeneration of skeletal muscle and inhibit fibrosis. **a** Representative image of regenerated quadriceps and gastrocnemius muscle stained for laminin at Day 28 after HLI. For quantification, the number of myofibers with central nuclei in five randomly selected fields in each ischemic muscle was counted. **b** Percentage of myofibers with central nuclei (**c**) Cross-sectional area (CSA) size of myofiber percentage of myofiber with central nuclei. Scale bars, 50 μm. *p < 0.05 versus the control; †*p* < 0.05 versus BM-MSC group; *n* = 5 per group. **d** Representative images from the experimental groups showing fibrosis after staining with Masson’s trichrome and CHP (green) in the ischemic muscle at Day 28 after HLI, and (**e**–**g**) the corresponding quantification results for (**e**) quantification summary of adipogenesis and (**f**) fibrosis. Scale bar, 100 μm. **g** Percentage of CHP-positive area. Scale bar, 20 μm, *p < 0.05 versus the control; †*p* < 0.05 versus BM-MSC group; *n* = 5 for each group. Data are presented as the mean ± SEM.

To further investigate whether SDF1α-eMSCs improved skeletal muscle regenerative efficacy in ischemic limbs, we quantified the main parameters of regenerating myofibers, which were the cross-sectional area (CSA) of the myofibers and the number of myofibers with central nuclei, by immunostaining for laminin. The CSA and the number of myofibers with central nuclei were significantly greater in the SDF1α-eMSC group than in the other groups (Fig. 5a–c). Subsequently, we also performed MT staining to quantify fibrosis and fat deposition 4 weeks after ischemia. Interestingly, we observed that fat deposition mainly occurred in the quadriceps muscle, whereas fibrosis was predominantly in the gastrocnemius muscle, a relatively severe ischemic site (Fig. 5d). Interestingly, intramuscular injection of SDF1α-eMSCs significantly reduced fat deposition in the quadriceps muscle and fibrosis in the gastrocnemius muscle compared with those of the other groups (Fig. 5e, f). Furthermore, the deposition of denatured collagen in the gastrocnemius muscle was significantly lower in the SDF1α-eMSC group than in the other groups (Fig. 5g). These findings suggested that SDF1α-eMSCs not only directly contribute to skeletal muscle regeneration by stimulating myogenesis but also provide a favorable microenvironment for skeletal muscle repair by reducing fibrosis and fat deposition.

### SDF1α-eMSCs also provided a reconstructive niche in the early stages of limb ischemia

**Fig. 6:**
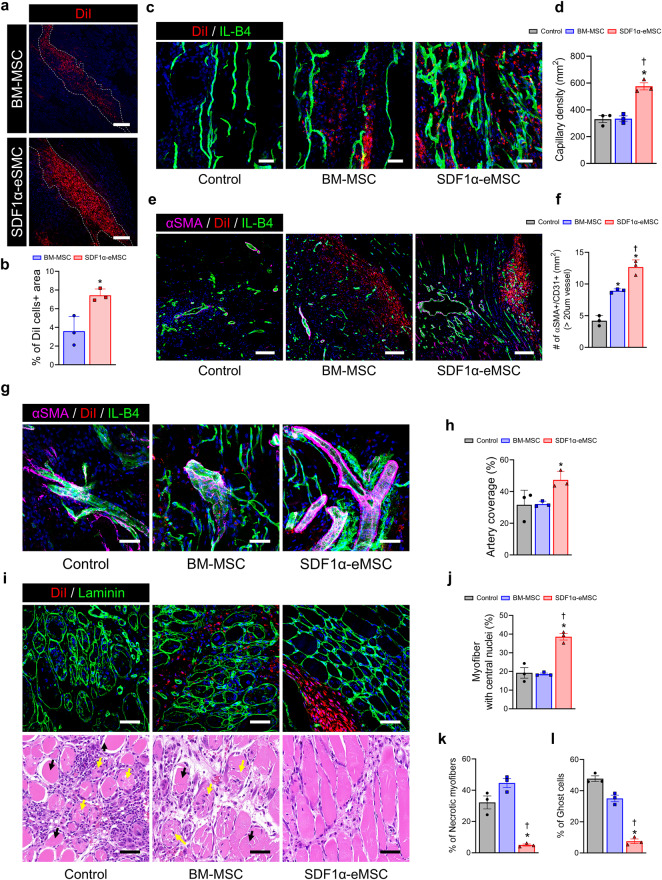
SDF1α-eMSCs simultaneously promote the regeneration of blood vessels and skeletal muscles at Day 7 after hindlimb ischemia. **a** Representative image of cells injected into the ischemic muscle on Day 7 after HLI and (**b**) the corresponding quantification summary. Scale bars, 200 μm. *p < 0.05 versus BM-MSC group; *n* = 3 for each group. **c** Representative image of capillaries stained for IL-B4 (green) and DiI cells in ischemic muscle at Day 7 after HLI, and (**d**) the corresponding quantification summary. Scale bars, 40 μm. *p < 0.05 versus the control; †*p* < 0.05 versus BM-MSCs; *n* = 3 for each group. **e** Representative image of arteries stained for IL-B4 (green), αSMA (violet) and DiI cells in ischemic muscle at Day 7 after HLI, and (**f**) the corresponding quantification summary. Scale bars, 100 μm. *p < 0.05 versus the control; †*p* < 0.05 versus BM-MSC group; *n* = 3 for each group. **g** Representative image of artery coverage stained for IL-B4 (green), αSMA (violet), DiI cells and DAPI for nuclei (blue) in ischemic muscle at Day 7 after HLI, and (**h**) the corresponding quantification summary. Scale bars, 50 μm. *p < 0.05 versus the control; †*p* < 0.05 versus BM-MSC group; *n* = 3 for each group. **i** Representative image of regeneration muscle stained for laminin (green), DiI cells and DAPI for nuclei (blue). Scale bar, 50 μm and representative image of necrotic myofibers and ghost cells stained with H&E. Scale bar, 50 μm, and (**j**–**l**) the corresponding quantification results for (**j**) the percentage of myofibers with central nuclei, (**k**) the percentage of necrotic myofibers, and (**l**) the percentage of ghost cells. *p < 0.05 versus the control; †*p* < 0.05 versus BM-MSC group; *n* = 3 for each group. Data are presented as the mean ± SEM.

Because intramuscular transplantation of SDF1α-eMSCs significantly improved blood perfusion and limb salvage beginning three days after ischemic insult, we addressed the role of SDF1α-eMSCs in the early stages of limb ischemia during which vascular remodeling and muscle regeneration occur using tissue harvested 7 days after Dil-labeled cell transplantation. First, the retention of transplanted cells within ischemic limb muscles was significantly greater in the SDF1α-eMSC group than in the BM-MSC group (Fig. 6a, b). Next, we performed isolectin B4 (ILB4) perfusion staining to identify perfused functional vessels. The number of ILB4^+^ perfused vessels was significantly greater in the SDF1α-eMSC group than in the other groups (Fig. 6c–f). Interestingly, the percentage of ILB4^+^ α-SMA^+^ arterioles was also significantly higher in the SDF1α-eMSC group than in the BM-MSC group, although Dil-labeled transplanted cells were not incorporated into the host vascular networks but were instead located in the perivascular area (Fig. 6g, h). These findings suggested that during the early stages of limb ischemia, SDF1α-eMSCs were involved mainly in the process of arterialization (defined as remodeling of preexisting collateral arteries to generate larger conductance vessels) but not in de novo vasculogenesis (defined as recruiting endothelial progenitor cells to foci of neovascularization where they form new blood vessels in situ).

**Fig. 7:**
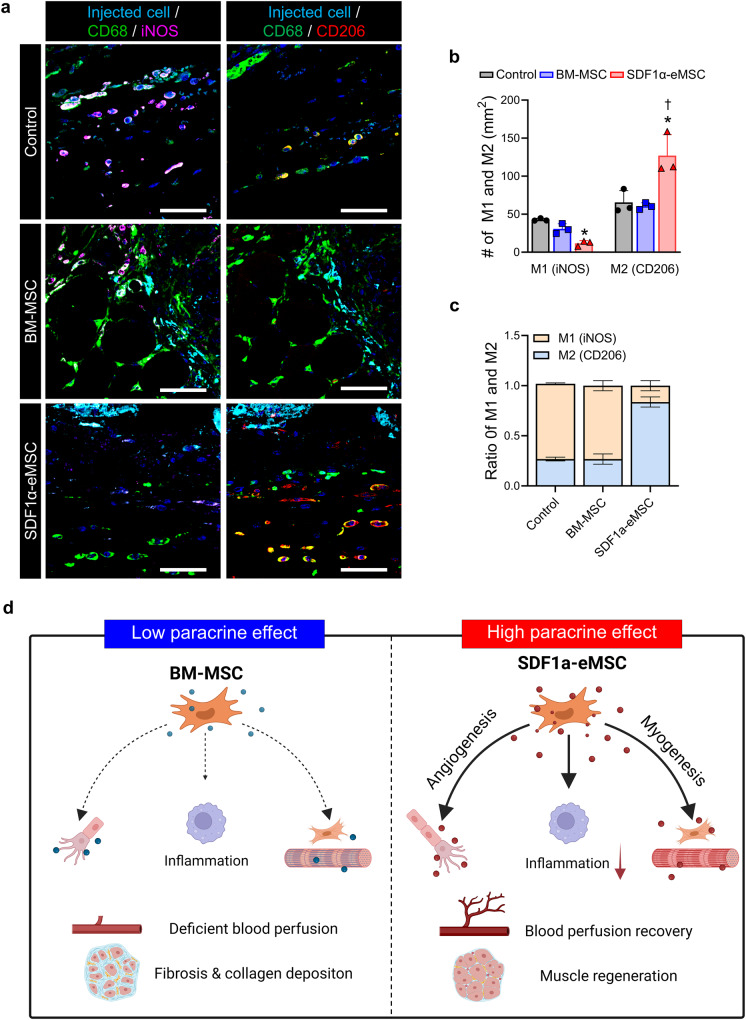
SDF1α-eMSCs positively regulate the inflammatory response in the ischemic muscle on Day 7 after hindlimb ischemia. **a** Representative image of proinflammatory macrophages (M1) and anti-inflammatory macrophages (M2) stained for injected cells (sky blue), CD68 (green), iNOS (purple), CD206 (red) and DAPI for nuclei (blue). Scale bars, 50 μm, and (**b**, **c**) the corresponding quantification summary. **b** The number of M1 and M2 macrophages; *p < 0.05 versus the control; †*p* < 0.05 versus BM-MSC group; *n* = 3 for each group. **c** M1:M2 ratio. Data are presented as the mean ± SEM. **d** Schematic diagram of the underlying therapeutic mechanisms of SDF1α-eMSCs.

Next, we investigated the role of SDF1α-eMSCs in skeletal muscle repair in ischemic limb tissue. Compared with the control group, the SDF1α-eMSC group had lower numbers of necrotic myofibers and ghost cells on H&E staining but a higher number of regenerated myofibers with central nuclei, as identified by immunostaining of laminin (Fig. 6i–l). These findings suggested that SDF1α-eMSCs not only protected against damage to skeletal muscles but also promoted their rapid repair after ischemic injury. Furthermore, we evaluated whether intramuscular SDF1α-eMSC transplantation could modulate the inflammatory response during the early stages of limb ischemia. We quantified the number of CD68^+^iNOS^+^ (M1) and CD68^+^CD206^+^ (M2) macrophages and the ratio of M1/M2 macrophages in ischemic limb tissues harvested 7 days after cell transplantation. The SDF1α-eMSC group had lower M1 and increased M2 macrophage numbers than the other groups (Fig. 7a, b). Additionally, M2 macrophages were more dominant in the SDF1α-eMSC group than in the other groups (Fig. 7c). Taken together, these findings suggest that in the mouse HLI model, SDF1α-eMSCs provided a favorable microenvironment for vascular regeneration and skeletal muscle repair in the early stages of limb ischemia by facilitating arterialization, modulating inflammation and protecting against skeletal muscle damage (Fig. 7d). These results provide compelling evidence that implantation of genetically engineered MSCs continuously secreting SDF-1α can be an effective strategy to achieve successful limb salvage in limb ischemia.

## Discussion

In the present study, we sought to develop an effective therapeutic strategy to simultaneously regenerate blood vessels and skeletal muscle in ischemic limbs by using SDF1α-eMSCs engineered to constantly secrete SDF-1α. The rationale behind this study was that despite intensive research and numerous attempts at therapeutic neovascularization for the treatment of PAD, functional limb salvage has not been obtained without the successful regeneration of skeletal muscles, another critical component of limb function. Nevertheless, most previous studies seeking to develop a new therapy for CLI have mainly focused on vascular regeneration rather than skeletal muscle regeneration^26^. We reasoned that even if neovascularization successfully restored blood flow following CLI, since the early regeneration potential of skeletal muscle cells was insufficient to achieve limb rejuvenation, it might ultimately lead to tissue necrosis and limb loss. Compared with previous reports, this study is, to the best of our knowledge, the first to successfully and concurrently regenerate both blood vessels and skeletal muscle in mice undergoing HLI.

We demonstrated that intramuscular injection of SDF1α-eMSCs significantly improved blood perfusion to ischemic limbs and preserved limb function without amputation in hindlimb ischemia (Fig. 3). Our in vitro results also support our findings that SDF1α-eMSCs prevented ECs from simulating ischemic insult and promoted EC migration and vessel formation, both of which are required for vascular regeneration, through increased phosphorylation of ERK and Akt within the SDF1α/CXCR4 axis (Fig. 1). Of interest, the SDF1α-eMSC group had a significantly greater arteriole (CD31 + αSMA + ) density within the ischemic limb 4 weeks after the ischemic insult, and such changes were evident even in the early stages of limb ischemia, as indicated by the greater percentages of ILB4+ α-SMA+ arterioles in the SDF1α-eMSC group than in the BM-MSC group (Fig. 4). More interestingly, our histological analyses demonstrated that the transplantation of SDF1α-eMSCs reduced the number of ghost cells and necrotic myofibers but enhanced the number of regenerated myofibers even at an early stage (one week post-ischemia; Fig. 6i–l). Furthermore, successful skeletal muscle regeneration mediated by SDF1α-eMSCs suppressed the deposition of denatured collagen, tissue fibrosis, and adipogenesis, resulting in improved limb salvage. Although early neovascularization induced by SDF1α-eMSCs could provide an indirect beneficial effect on skeletal muscles, our in vitro experiments further revealed that the paracrine factors secreted by SDF1α-eMSCs protected myoblasts and promoted their migration under cellular starvation conditions simulating ischemia through the ERK and Akt pathways within the SDF1α/CXCR4 axis. These findings were consistent with a previous report showing that increased phosphorylation of Akt enhanced myoblast migration and differentiation into skeletal muscle^27^. (Fig. 2). Finally, with respect to another crucial underlying tissue protective mechanism, we found that SDF1α-eMSCs recruited a significantly greater number of M2 anti-inflammatory macrophages than M1 proinflammatory macrophages for up to 7 days after ischemic insult (Fig. 7a–c). Giri J et al. also reported that the BM-MSC-secreted chemokine CXCL12 upregulated IL-10 expression in CCR2^+^ macrophages, which could lead to M2 polarization and be involved in anti-inflammatory responses^28^. These results are consistent with our premise that SDF1α-eMSCs positively modulated the hostile microenvironment in a similar fashion by regulating the inflammatory response in hindlimb ischemic tissues.

While SDF-1α has been actively pursued as a potential target for drug development because it can regulate multiple cellular processes, such as cell migration and survival, angiogenesis, and immune responses, its extremely short half-life (26 min in vivo) renders its therapeutic effects as a chemokine ineffective^29^. In particular, the therapeutic efficacy of the SDF-1α chemokine could be less effective within the ischemic microenvironment in vivo due to many cytokine-binding proteases, inhibitors and soluble cytokine receptors that can interfere with the effect of SDF-1α^30^. To overcome this fundamental limitation and to revisit the therapeutic potential of SDF-1α, we genetically modified MSCs derived from human bone marrow to be able to release SDF-1α continuously (SDF1α-eMSCs) and injected them into the ischemic hindlimb to address their therapeutic efficacy and safety concerns. Notably, we found that SDF1α-eMSCs exhibited substantially greater retention and engraftment rates than BM-MSCs in ischemic limb tissues on Day 7 after transplantation, resulting in increased numbers of functional vessels as well as regenerated myofibers, which could induce complete blood flow recovery and functional limb salvage. These findings suggest that cell therapeutics using genetically engineered SDF1α-eMSCs can be a suitable strategy to achieve functional limb salvage while also preventing undesirable side effects in vivo.

In summary, we demonstrated that SDF1α-eMSCs induced concurrent angiogenesis and myogenesis, which together could lead to significant functional limb salvage from ischemic injury through enhanced paracrine actions mediated by SDF-1α. Our results indicate that SDF1α-eMSCs may be a suitable option for cell-based therapy to treat patients undergoing CLI, for which few treatment options are currently available.

### Supplementary information

Supplementary information
