# Supplementary material for: Vascular regeneration and skeletal muscle repair induced by long-term exposure to SDF-1α derived from engineered mesenchymal stem cells after hindlimb ischemia
Source: Exp Mol Med. 2023 Oct 2;55(10):2248–59. doi: 10.1038/s12276-023-01096-9 (PMC10618463; doi:10.1038/s12276-023-01096-9)
Supplement: Supplementary file 1 — Supplementary information [file 12276_2023_1096_MOESM1_ESM.pdf]

Supplementary information

Vascular regeneration and skeletal muscle repair induced by long-term exposure to SDF-1α derived from engineered mesenchymal stem cells after hindlimb ischemia

Jin-Ju Kim[1,2,], Jae-Hyun Park[1,2], Hyeok Kim[1,2], Woo-Sup Sim[1,2], Seokbeom Hong[3], Yeon-Jik Choi[4], Hyo-Jin Kim[5], Soon Min Lee[5], Dongha Kim[7], Sun-woong Kang[8], Kiwon Ban[6,#], Hun-Jun Park[1,2,#]

[1] Department of Biomedicine & Health Sciences, The Catholic University of Korea,

[2] Division of Cardiology, Department of Internal Medicine, Seoul St. Mary's Hospital, College of Medicine, The Catholic University of Korea,

[3] Department of Thoracic and Cardiovascular Surgery, Seoul St. Mary's Hospital, College of Medicine, The Catholic University of Korea

[4] Division of Cardiology, Department of Internal Medicine, Eunpyeong St. Mary's Hospital, College of Medicine, The Catholic University of Korea

[5] SL BIGEN, Inc.

[6] Department of Biomedical Sciences, City University of Hong Kong,

[7] Research Group for Biomimetic Advanced Technology, Korea Institute of Toxicology 7 (KIT), Daejeon 34114, Republic of Korea

[8] Department of Anatomy, College of Medicine, The Catholic University of Korea

# Address for Correspondence:

Hun-Jun Park, MD, PhD, Seoul St. Mary's Hospital, The Catholic University of Korea, 222 Banpo-daero, Seocho-gu, Seoul, 137-701, Republic of Korea. Email: cardioman@catholic.ac.kr or Kiwon Ban, PhD, City University of Hong Kong, 83 Tat Chee Avenue, Kowloon, Hong Kong SAR. E-mail: kiwonban@cityu.edu.hk

Supplementary information

Supplementary figures

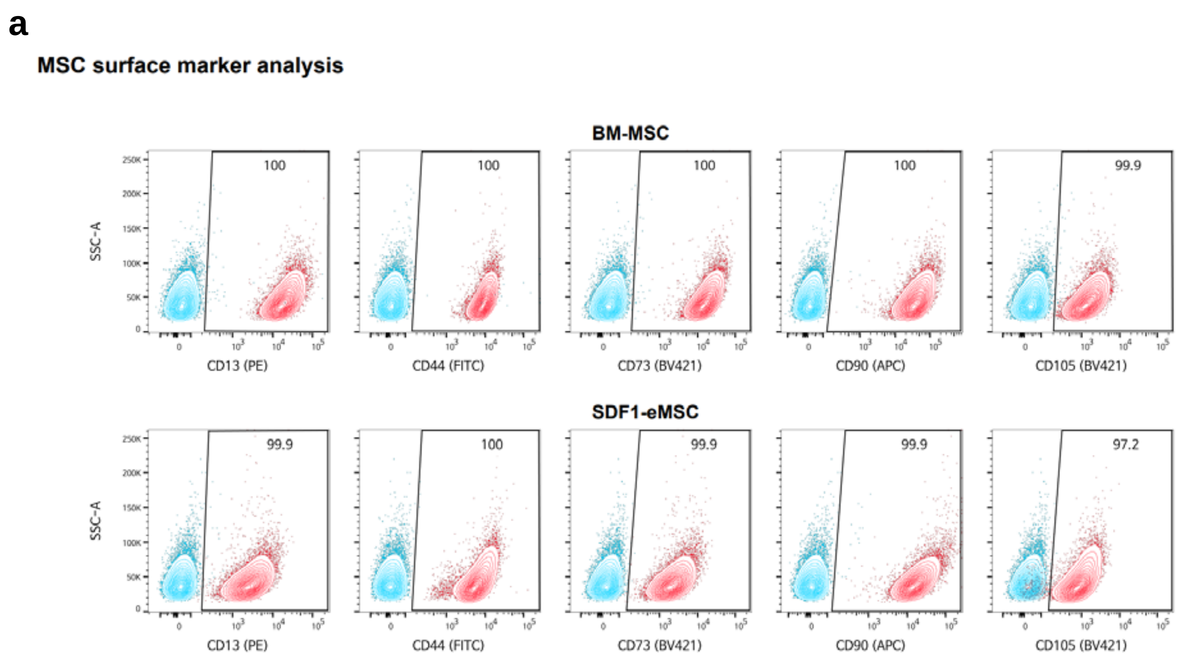

Supplementary Fig 1. Cellular characterization of SDF1α-eMSCs

MSC surface marker expression. **a** MSC-related surface markers expressed on BM-MSCs. **b** MSC-related surface markers expressed on SDF1α-eMSCs

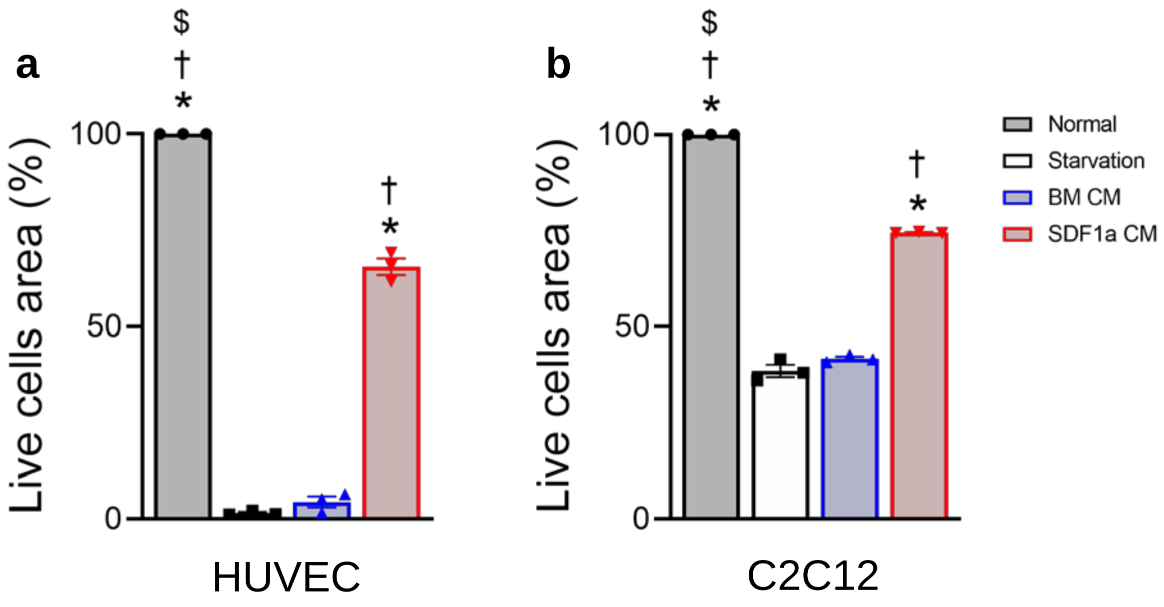

Supplementary Fig 2. Cytoprotective effects of SDF1α against starvation simulating ischemic insult.

Cell viability using a fluorescent LIVE/DEAD™ Viability assay. Green fluorescence (calcein-AM) indicates live cells, and red fluorescence (ethidium homodimer-1) indicates dead cells. **a** Area of live HUVEC cells **b** Area of live C2C12 cells after 24 h in serum-free media. Scale bars =100 μm. * $p < 0.05$ versus starvation group; † $p < 0.05$ versus BM group; $ $p < 0.05$ versus SDF group; $n = 3$ for each group. Data are presented as the mean ± SEM.

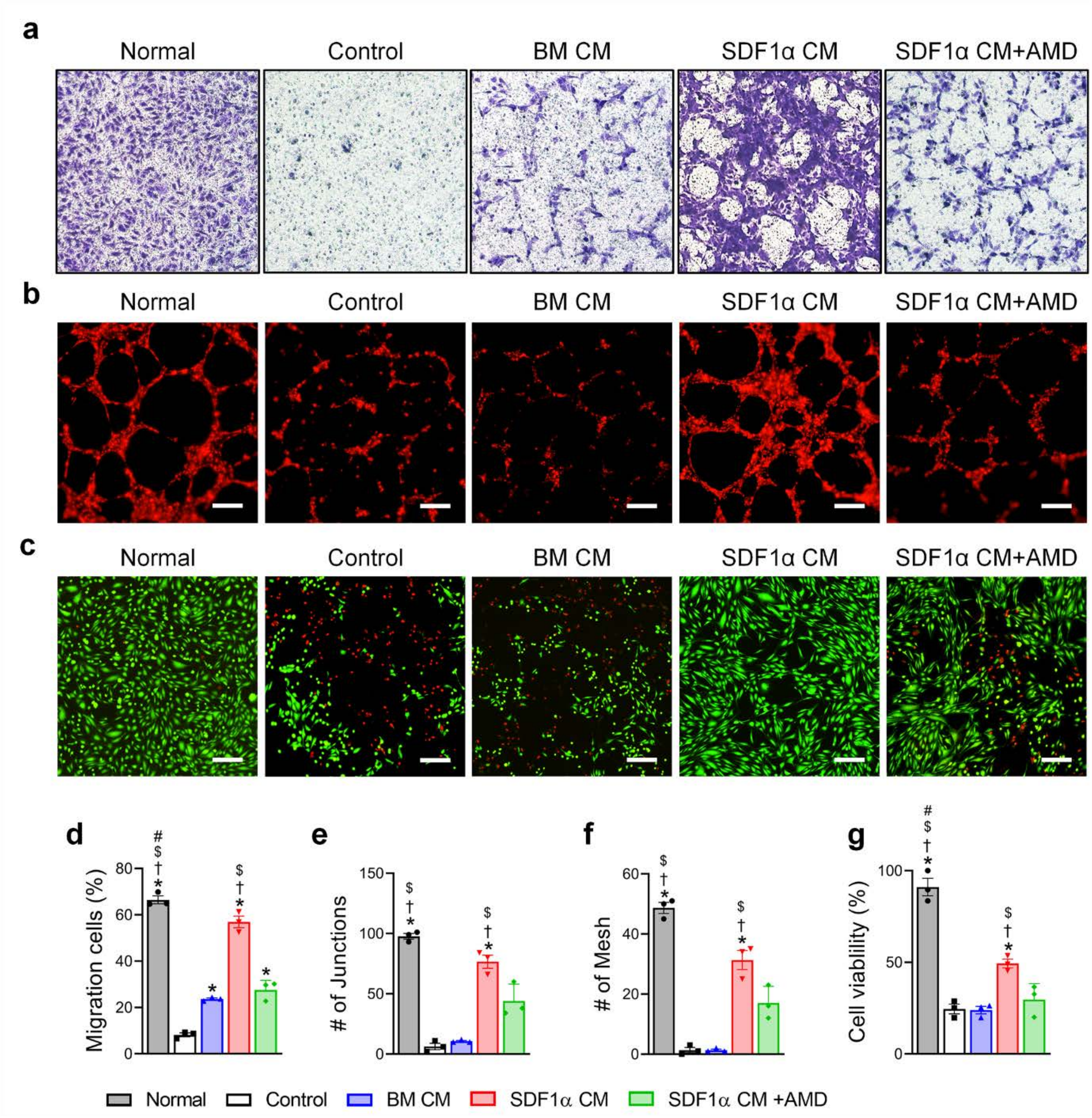

Supplementary Fig 3. AMD3100 treatment block the SDF1a CM effect of cells function in HUVEC

a Migration assay **b** Tube formation. Scale bars =200µm. **c** LIVE/DEAD™ Viability assay. Scale bars =200µm. **d** Statistical analysis of migration cells**. e-f** Statistical analysis of tube formation assay. **g** Statistical analysis of LIVE/DEAD™ Viability assay. Quantification summary is shown. *$p < 0.05$ versus Control; †$p < 0.05$ versus BM CM; \$$p < 0.05$ versus SDF1α CM+AMD; #$p < 0.05$ versus SDF1α CM; $n = 3$ per group. Data are presented as the mean ± SEM.

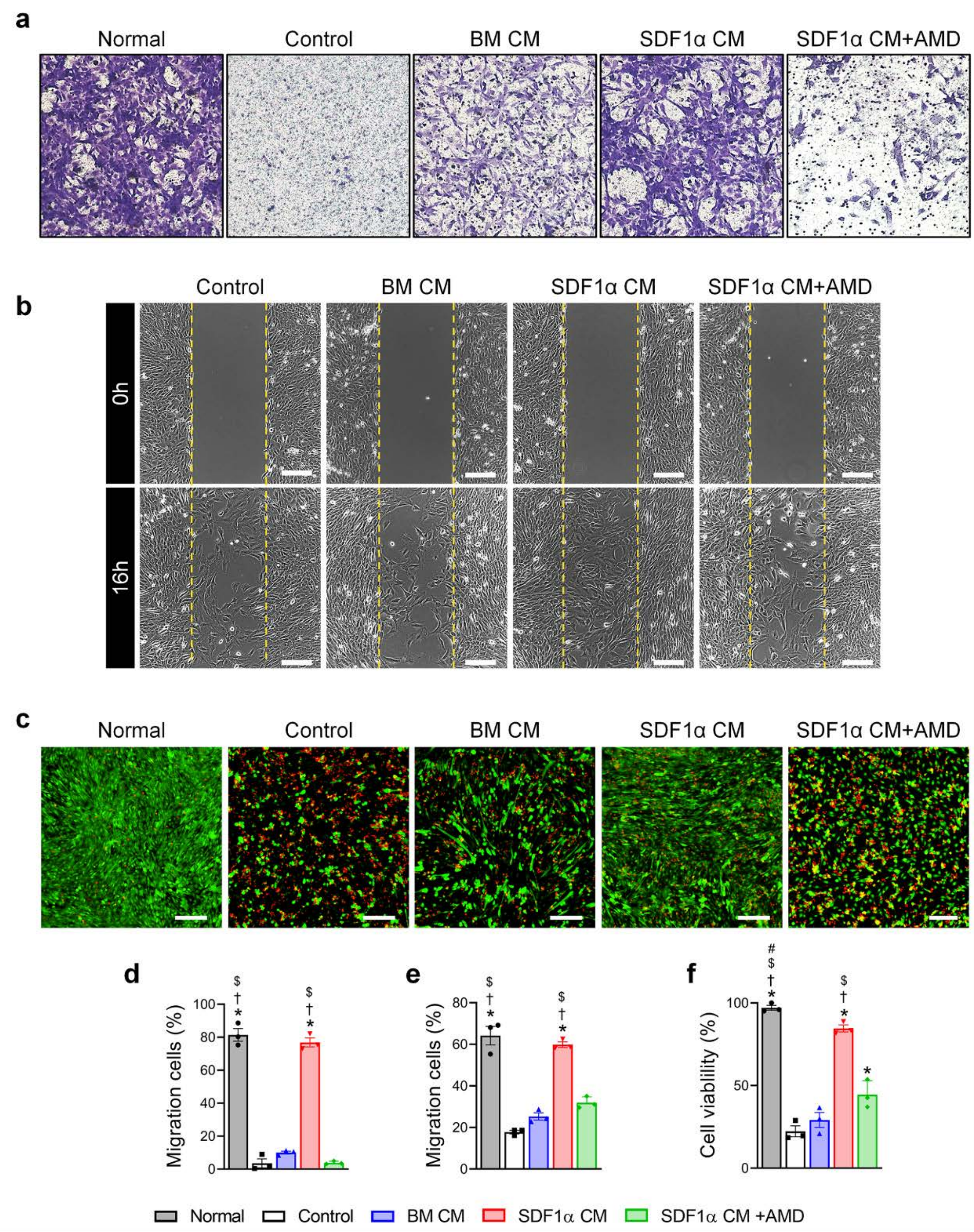

Supplementary Fig 4. Effect of the SDF1a paracrine was offset by AMD3100 in C2C12

a Migration assay **b** Scratch assay. Scale bars =200μm. **c** LIVE/DEAD™ Viability assay. Scale bars =200μm. **d** Statistical analysis of migration cells**. e** Statistical analysis of scratch assay. **f** Statistical analysis of LIVE/DEAD™ Viability assay. Quantification summary is shown. *$p < 0.05$ versus Control; †$p < 0.05$ versus BM CM; \$$p < 0.05$ versus SDF1α CM+AMD; #$p < 0.05$ versus SDF1α CM; $n = 3$ per group. Data are presented as the mean ± SEM.

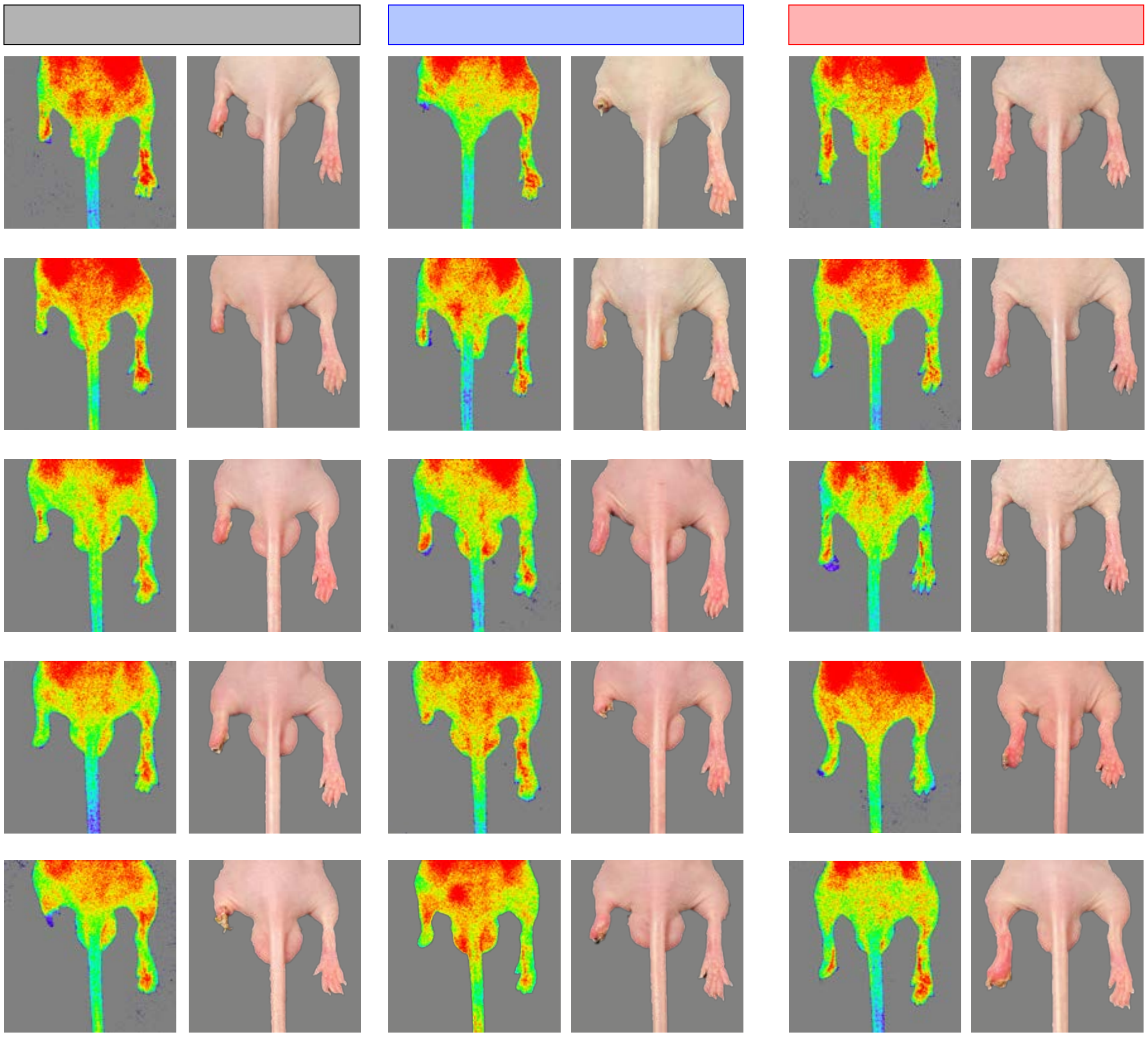

Supplementary Fig 5. Laser Doppler image and optical image of all group

a Laser Doppler perfusion imaging (LDPI) and optical image of ischemic hindlimb at Day 28 after implantation
